# Annalist

**Published:** 1849-08

**Authors:** 


*Annalist.*—This Journal has been discontinued in consequence of the Editor, Dr. N. S. Davis, having accepted the appointment of Professor of Physiology in the Rush Medical College, Chicago, Illinois, and his removal to that city as a permanent resident. We trust the professorial labors of Dr. D. will not preclude his continued contributions to periodical medical literature.

